# hsa_circ_0006916 promotes hepatocellular carcinoma progression by activating the miR-337-3p/STAT3 axis

**DOI:** 10.1186/s11658-020-00238-5

**Published:** 2020-11-02

**Authors:** Xiao-Yong Zhou, Hui Yang, Yang-Qiu Bai, Xiu-Ling Li, Shuang-Yin Han, Bing-Xi Zhou

**Affiliations:** Department of Gastroenterology and Hepatology, Henan Provincial People’s Hospital, People’s Hospital of Zhengzhou University, No. 7 Weiwu Road, Jinshui District, Zhengzhou, 450003 Henan China

**Keywords:** hsa_circ_0006916, miR-337-3p, STAT3, Hepatocellular carcinoma

## Abstract

**Background:**

Circular RNAs (circRNAs) are thought to be involved in the development of various malignancies. The expression and function of hsa_circ_0006916, a newly identified circRNA, in hepatocellular carcinoma remain unclear.

**Methods:**

Quantitative RT-PCR was used to detect hsa_circ_0006916 in hepatocellular carcinoma. In vitro function assays were conducted to explore growth and invasion of hepatocellular carcinoma cells. Next, the mechanism of hsa_circ_0006916 function in hepatocellular carcinoma was determined by luciferase reporter and RIP assays.

**Results:**

Hsa_circ_0006916 was substantially overexpressed in hepatocellular carcinoma tissues and cells. High levels of hsa_circ_0006916 in hepatocellular carcinoma patients were associated with advanced clinical characteristics. Down-regulation of hsa_circ_0006916 decreased the growth and invasion of hepatocellular carcinoma cells in vitro. The results suggested that hsa_circ_0006916 acted as a sponge of miR-337-3p and had an important functional use in the regulation of STAT3 levels in hepatocellular carcinoma cells. Moreover, miR-337-3p inhibition or STAT3 overexpression abolished the effect of hsa_circ_0006916 suppression on the progression of hepatocellular carcinoma cells.

**Conclusions:**

Our data suggest a novel hsa_circ_0006916/miR-337-3p/STAT3 axis in hepatocellular carcinoma, and provide a new target for treatment.

## Introduction

Hepatocellular carcinoma is a common malignant disease worldwide with a high mortality rate [1, 2]. Although significant progress has been made in the field of hepatocellular carcinoma over the past decades, the prognosis remains inadequate because of a high recurrence and metastasis rate [3, 4]. Thus, it is crucial to determine the mechanism behind hepatocellular carcinoma tumorigenesis.

Increasing data have shown that non-coding RNAs (ncRNAs) have a critical function in post-transcriptional gene regulation of different diseases [5, 6]. Circular RNA (circRNA) is a form of RNA composed of a covalent closed-loop structure, no 5′-3′ polarity, and no polyadenylated tail [7, 8]. The latest reports show that circRNAs were abnormally expressed in various cancers and play vital roles in progression of malignancies [9, 10]. For example, the expression of circRNA_10223 was found to be elevated in lung carcinoma and correlated with tumor progression [11]. circRNA_069718 was discovered to promote the progression of breast cancer via regulating the Wnt/β-catenin axis [12].

Here, we identified a new circular RNA (hsa_circ_0006916) in hepatocellular carcinoma. Our results are the first to reveal that the growth and invasion of hepatocellular carcinoma cells were improved by highly expressed hsa_circ_0006916. Regarding the mechanism, the data suggested that hsa_circ_0006916 may act as a sponge of miR-337-3p and play a vital role in upregulating STAT3 levels. Our data may provide experimental evidence for potential hepatocellular carcinoma therapy.

## Material and methods

### Human tissue samples

Overall, 59 cases of hepatocellular carcinoma and matched adjacent non-tumor tissues (ANT; > 3 cm away from the cancerous tissue) were acquired from the Henan Provincial People’s Hospital from June 2017 to January 2018. The experimental protocols were granted approval by the ethics committee of our hospital.

### Materials and cell lines

Five human hepatocellular carcinoma (Hep3B, Bel-7404, Huh7, HepG2 and SMMC-7721) and one healthy human liver (HL-7702) cell lines were acquired from the Chinese Academy of Sciences (Beijing, China), and sustained in DMEM (Invitrogen, USA) supplied with 10% FBS (Invitrogen, USA), 1% penicillin (Sigma, Italy) or streptomycin (Sigma, Italy) at 37 °C, 5% CO_2_.

siRNAs targeting hsa_circ_0006916 (si-circ_0006916#1/2), STAT3 overexpression plasmids, miR-337-3p mimics and inhibitors, and scramble controls were obtained from Genechem (Shanghai, China) and transfected into hepatocellular carcinoma cells according to the instructions of Lipofectamine 3000 (Invitrogen, USA).

### Subcellular fractionation assay

Cytoplasmic and nuclear RNA were isolated through the Cytoplasmic and Nuclear RNA Purification Kit (Norgen, Canada) in accordance with the product’s guidelines. Relative changes in gene expression were measured by quantitative RT-PCR (RT-qPCR), which was conducted using previously published methods [12].

### 5-Ethynyl-2′-deoxyuridine (EdU) assay

The growth of hepatocellular carcinoma cell was evaluated using an EdU kit (Roche, Germany). Cells (2 × 10^3^ cells/well) were incubated with EdU solution, supplemented with 4% paraformaldehyde for half an hour, Triton X-100 was added to each well, and then the cells were stained with Apollo solution. Finally, cell nuclei were stained with DAPI (Sigma, USA).

### Colony formation assay

Transfected hepatocellular carcinoma cells in DMEM medium were seeded onto 6-well plates. After 2 weeks, cell colonies were fixed, stained and eventually counted through ImageJ software.

### Transwell assay

A total of 1 × 10^5^ transfected cells were dissolved in 0.2 ml of serum-free cell culture, and transplanted into chambers which were precoated with Matrigel (BD Biosciences, USA). Next, cells were stained with 0.1% crystal violet after 1 day. The quantity of hepatocellular carcinoma cells that had invaded through the Matrigel were measured under a microscope.

### Luciferase reporter assay

The wild-type (Wt) and mutant (Mut) sites of miR-337-3p binding on hsa_circ_0006916 or STAT3 3′UTR were sub-cloned into a dual-luciferase vector (pmirGLO). According to the instructions of the detection kit, the plasmids and miR-337-3p mimics were co-transfected into HEK293 cells and co-incubated for 48 h. Next, the luciferase activity was evaluated using a luciferase detection kit (Promega, USA).

### RNA immunoprecipitation (RIP) assay

The RIP assay was carried out by the method detailed in a previous report [13].

### Statistical analysis

SPSS 20.0 was applied for analyzing data. The measurement data were represented as means ± SD. Student’s t-test or one-way ANOVA was carried out for comparison between different groups. p < 0.05 represents statistical significance.

## Results

### Hsa_circ_0006916 is highly expressed in hepatocellular carcinoma

**Fig. 1:**
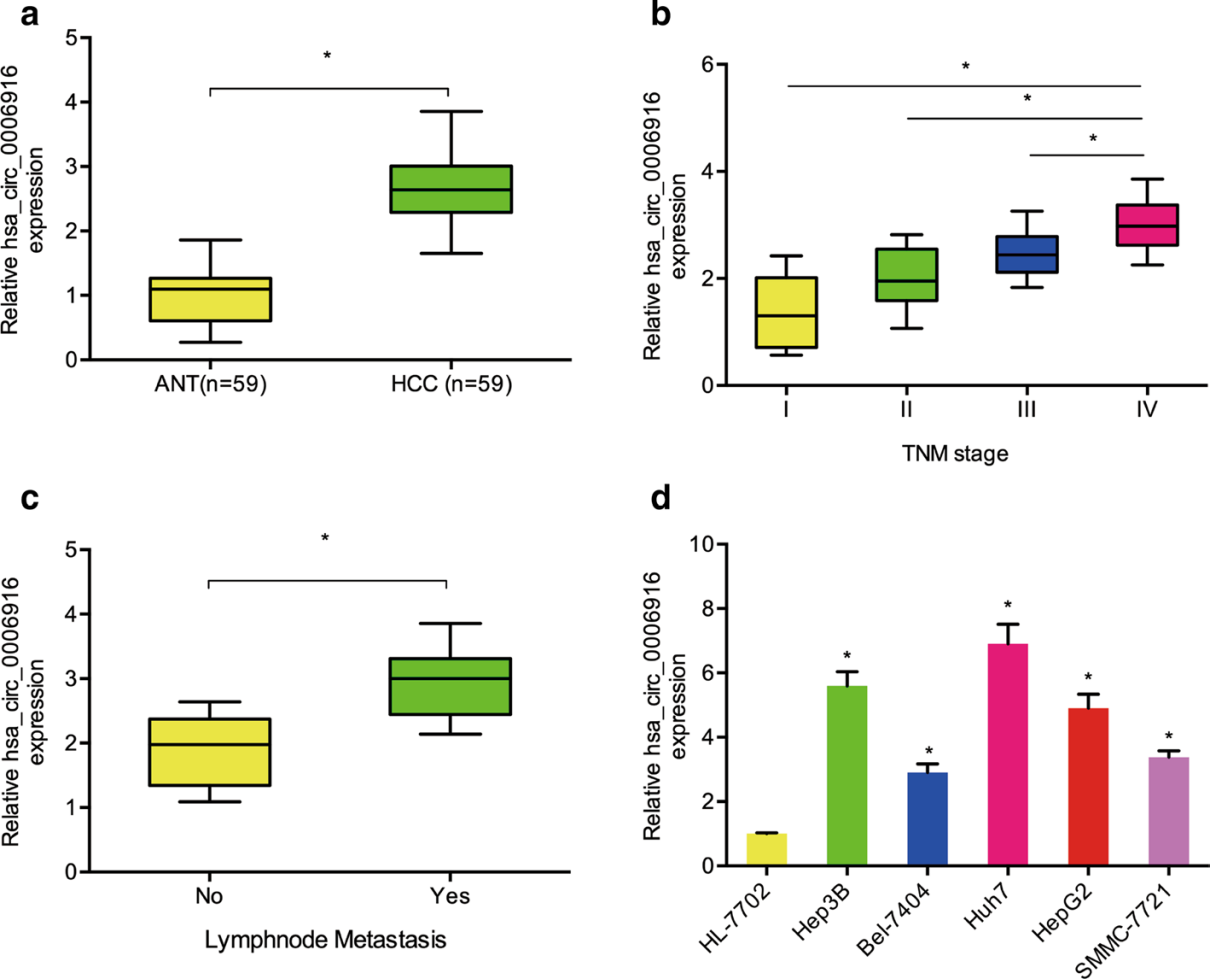
Hsa_circ_0006916 is upregulated in hepatocellular carcinoma. **a** Hsa_circ_0006916 levels in adjacent normal tissues (ANT) and hepatocellular carcinoma (HCC) tissues. **b** Hsa_circ_0006916 levels in hepatocellular carcinoma at different TNM stages. **c** Hsa_circ_0006916 levels in hepatocellular carcinoma with or without lymph node metastasis. **d** Hsa_circ_0006916 levels in hepatocellular carcinoma cell lines. *p < 0.05

Recently, Xu et al*.* demonstrated that hsa_circ_0006916 is one of the highest expressed circRNAs in hepatocellular carcinoma [13]. Nevertheless, the effect and underlying mechanism of hsa_circ_0006916 in hepatocellular carcinoma remain largely unknown. We demonstrated that hsa_circ_0006916 was notably upregulated in hepatocellular carcinoma patients, and correlated with clinical pathological features, including advanced TNM stage and lymph node metastasis (Fig. 1a–c). In addition, qRT-PCR results showed aberrant overexpression of hsa_circ_0006916 in Hep3B, Bel-7404, Huh7, HepG2 and SMMC-7721 (Fig. 1d). Hence, hsa_circ_0006916 is suggested to be involved in the development of hepatocellular carcinoma.

### Hsa_circ_0006916 promoted growth and invasion of hepatocellular carcinoma cells

**Fig. 2:**
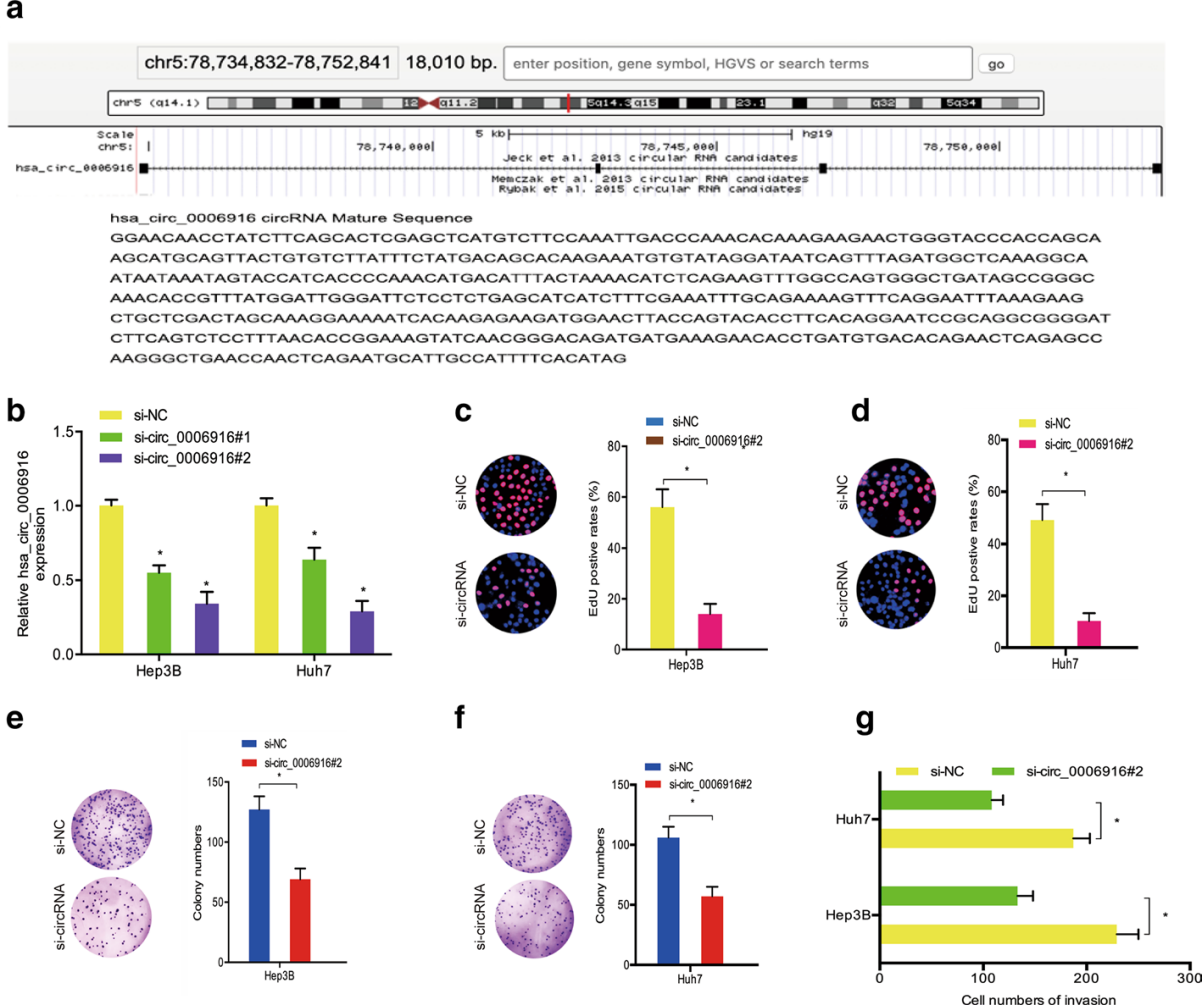
Knockdown of hsa_circ_0006916 reduced hepatocellular carcinoma advancement. **a** Hsa_circ_0006916 data. **b** Knockdown efficacy of si-circ_0006916 using qRT-PCR. **c**–**f** EdU and colony formation experimental results for the growth of hepatocellular carcinoma upon knockdown of hsa_circ_0006916. **g** Transwell assay determined the invasion of hepatocellular carcinoma cells transfected with si-circ_0006916. *p < 0.05

To elucidate the effect of hsa_circ_0006916 on hepatocellular carcinoma, we first identified the genomic location of hsa_circ_0006916 through UCSC, which was at chr5q14.1 (Fig. 2a). Then, we transfected si-circ_0006916 and si-NC into Hep3B and Huh-7 cells (Fig. 2b). EdU and colony formation experiments indicated that hsa_circ_0006916 inhibition reduced the growth of Hep3B and Huh-7 cells in vitro (Fig. 2c–f). In addition, results from the transwell assay demonstrated that hsa_circ_0006916 reduction led to a decrease in the invasive ability of Hep3B and Huh-7 cells in vitro (Fig. 2g).

### Hsa_circ_0006916 interacted with miR-337-3p in hepatocellular carcinoma

**Fig. 3:**
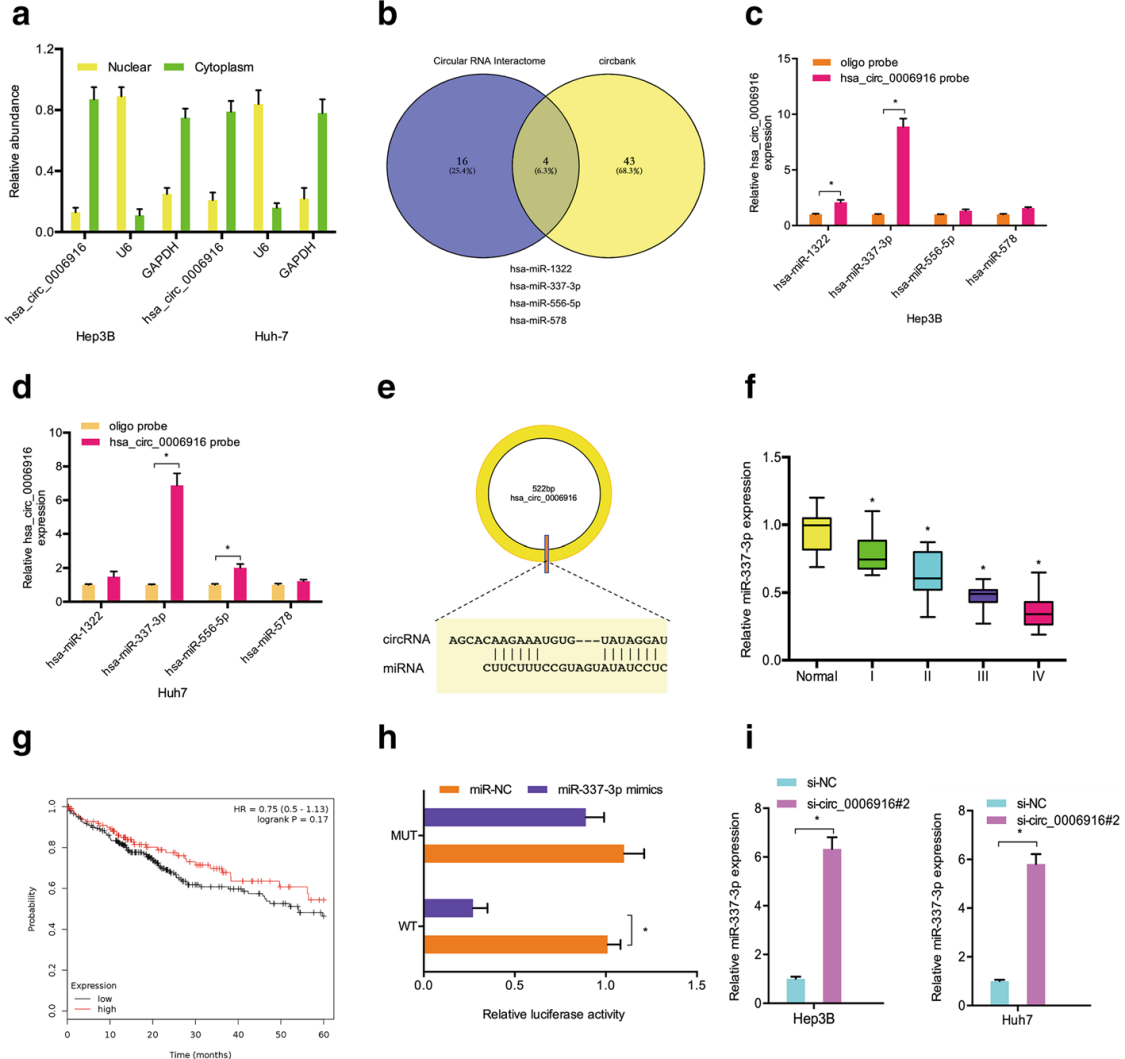
MiR-337-3p is directly targeted by hsa_circ_0006916. **a** Location of hsa_circ_0006916 in hepatocellular carcinoma cells. **b** Potential targets of hsa_circ_0006916. **c**, **d** RNA pull-down assay demonstrated that miR-337-3p was highly expressed in hsa_circ_0006916 probe-biotin. **e** Binding sites between hsa_circ_0006916 and miR-337-3p. **f** miR-337-3p in hepatocellular carcinoma and adjacent normal samples. **g** Reduced miR-337-3p levels related to poor prognosis. **h** miR-337-3p mimics decreased the luciferase activity of hsa_circ_0006916-WT group. **i** hsa_circ_0006916 inhibition upregulated miR-337-3p in hepatocellular carcinoma cells. **j** Hsa_circ_0006916 and miR-337-3p levels have a negative correlation in hepatocellular carcinoma. *p < 0.05

Increasing studies have revealed that circRNAs in the cytoplasm regulate gene expression by sponging miRNAs [14, 15]. In the current research, we determined the position of hsa_circ_0006916 in vitro. Subcellular fractionation analysis confirmed the cytoplasmic localization of hsa_circ_0006916 in Hep3B and Huh-7 cells (Fig. 3a). Venn diagram analysis showed that 4 miRNAs (miR-1322, miR-337-3p, miR-556-5p, miR-578) had binding sites for hsa_circ_0006916 (Fig. 3b). Through RNA pull-down assay, it was found that accrual of miR-337-3p was the highest in the hsa_circ_0006916 probe-biotin group (Fig. 3c–e). Next, miR-337-3p was found to be reduced in tumor tissues, which was related to poor prognosis (Fig. 3f, g). The luciferase reporter assay demonstrated that miR-337-3p mimics decreased the luciferase activity of the hsa_circ_0006916-WT group (Fig. 3h). Hsa_circ_0006916 inhibition significantly increased miR-337-3p in Hep3B and Huh-7 cells (Fig. 3i). Moreover, we revealed the correlation of hsa_circ_0006916 and miR-337-3p levels in hepatocellular carcinoma (Fig. 3j). In sum, these data indicate that hsa_circ_0006916 might interact with miR-337-3p in hepatocellular carcinoma.

### MiR-337-3p targeted STAT3 in hepatocellular carcinoma

**Fig. 4:**
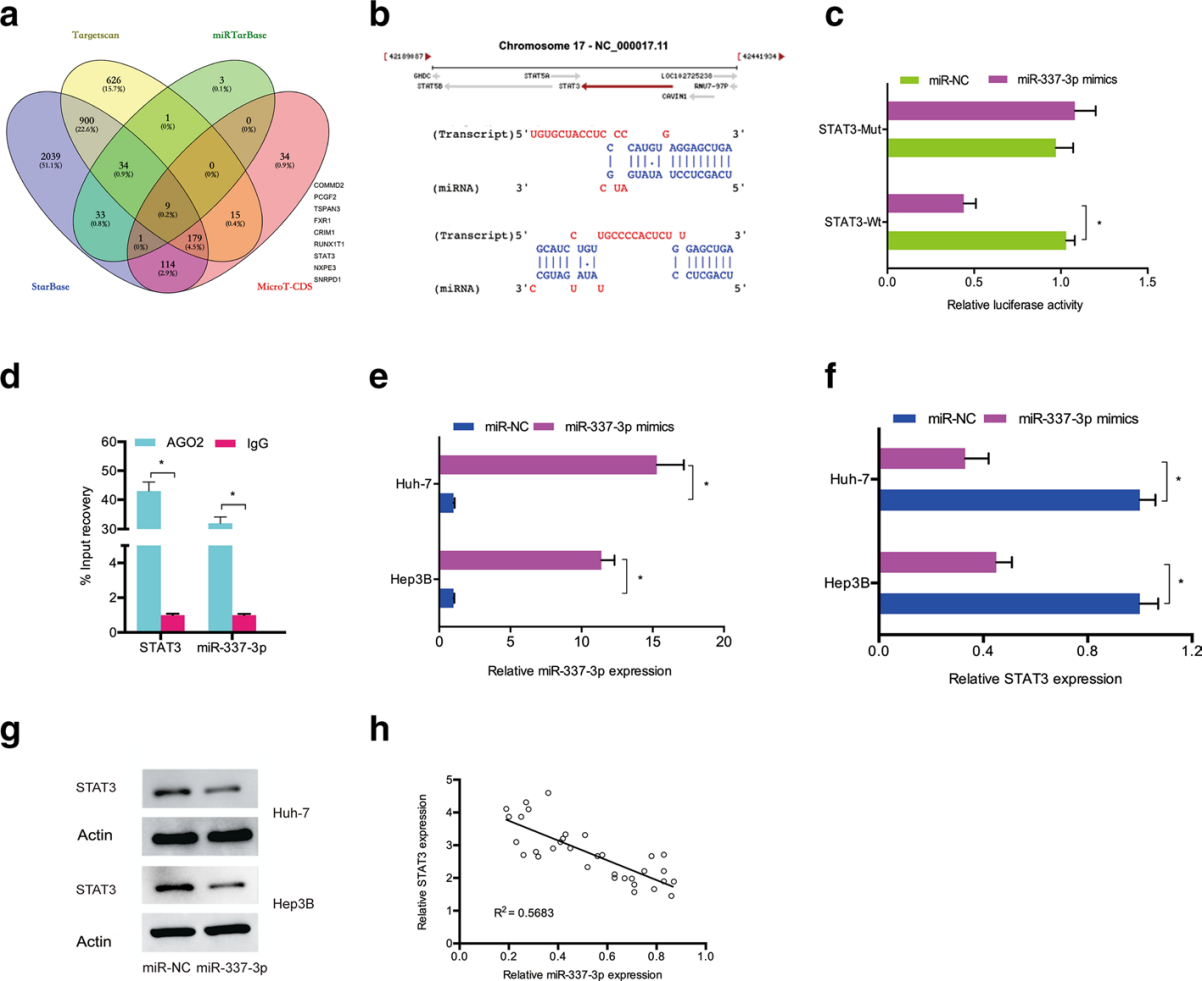
STAT3 is targeted by miR-337-3p. **a** Identification of miR-337-3p’s target genes by 4 databases. **b** Predicted binding sites between miR-337-3p and STAT3. **c** miR-337-3p mimics attenuated the luciferase activity of STAT3-WT group. **d** RIP assay was used to validate the correlation between miR-337-3p and STAT3. **e** Transfection efficiency of miR-337-3p mimics was explored by qRT-PCR. **f**, **g** MiR-337-3p mimics reduced STAT3 expression in hepatocellular carcinoma cells. **h** MiR-337-3p was negatively correlated with STAT3 in hepatocellular carcinoma. *p < 0.05

Next, we evaluated the downstream miR-337-3p targets, and bioinformatics analysis (TargetScan, StarBase, miRTarBase, and MicroT-CDS) indicated that STAT3 might be a putative target (Fig. 4a, b). The luciferase reporter experiment revealed that miR-337-3p mimics attenuated the luciferase activity of the STAT3-WT group (Fig. 4c). The RIP assay validated the augmentation of miR-337-3p and STAT3 mRNA in the precipitates of anti-Ago2 (Fig. 4d). In addition, we confirmed that miR-337-3p was upregulated by miR-337-3p mimics in Hep3B and Huh-7 cells (Fig. 4e). QRT-PCR and western blotting demonstrated that miR-337-3p mimics substantially decreased STAT3 levels in Hep3B and Huh-7 cells (Fig. 4f, g). Moreover, correlational analysis suggested that miR-337-3p levels were inversely related to STAT3 levels in hepatocellular carcinoma (Fig. 4h).

**Fig. 5:**
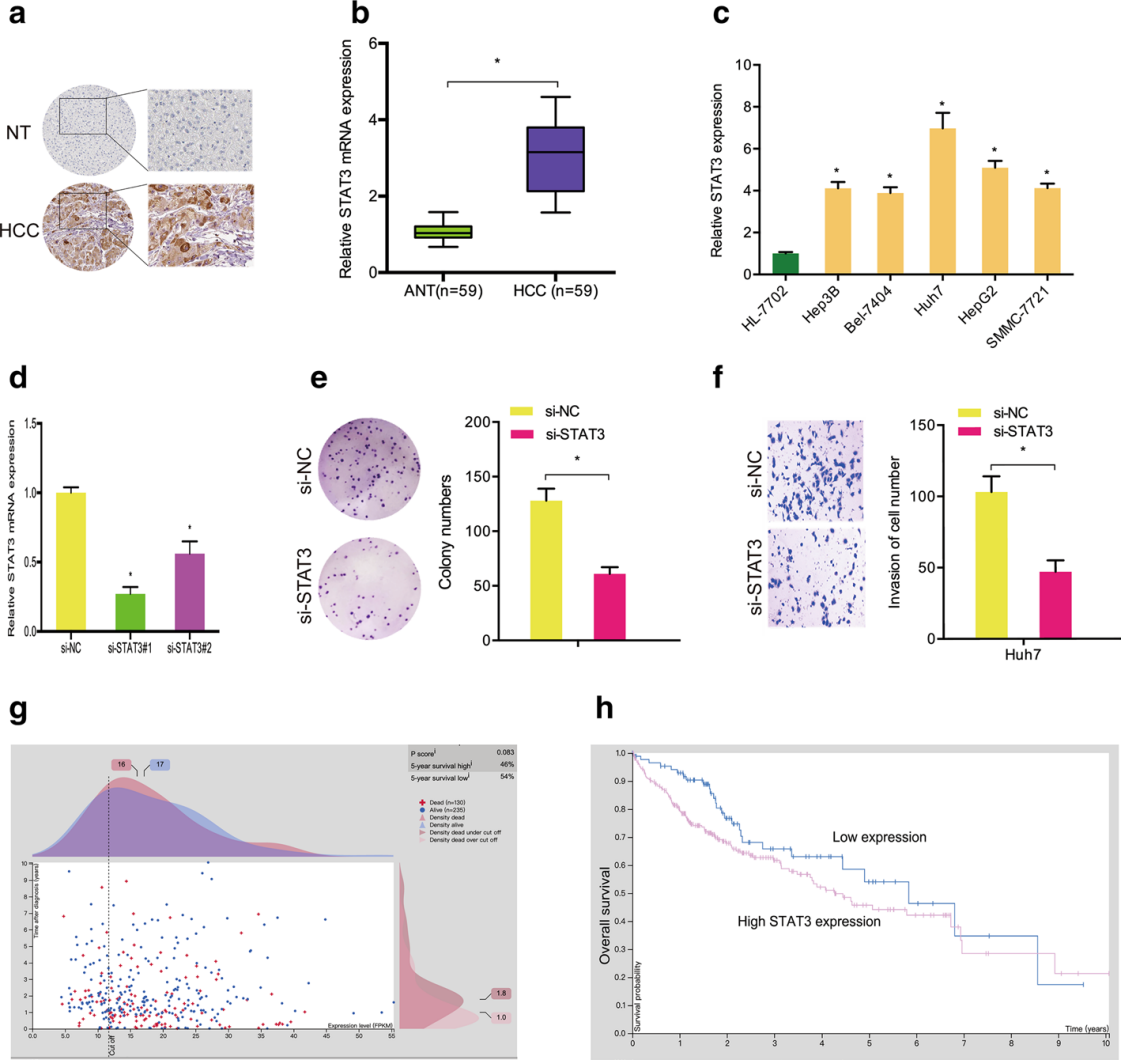
Function of STAT3 in hepatocellular carcinoma. **a** STAT3 in hepatocellular carcinoma was evaluated by IHC. **b** STAT3 in hepatocellular carcinoma tissue was explored by qRT-PCR. **c** STAT3 in hepatocellular carcinoma cell lines. **d** Transfection efficiency of si-STAT3 in Huh7 cells. **e**, **f** STAT3 inhibition reduced Huh7 cell proliferation and invasion abilities. **g**, **h** High STAT3 levels were correlated with poor prognosis in hepatocellular carcinoma patients. *p < 0.05

Next, we explored the roles of STAT3 in hepatocellular carcinoma. Immunohistochemistry (IHC) demonstrated that STAT3 was substantially overexpressed in hepatocellular carcinoma tissues (Fig. 5a). The results were further confirmed by qRT-PCR (Fig. 5b). Subsequently, we showed that STAT3 was increased in five hepatocellular carcinoma cells compared with one healthy human liver cell (Fig. 5c). Colony formation and transwell assays demonstrated that downregulation of STAT3 decreased Huh7 cell proliferation and invasion abilities in vitro (Fig. 5d–f). Moreover, our data showed that high STAT3 expression in hepatocellular carcinoma patients was correlated with shorter overall survival (Fig. 5g, h).

### Hsa_circ_0006916/miR-337-3p/STAT3 axis in hepatocellular carcinoma

**Fig. 6:**
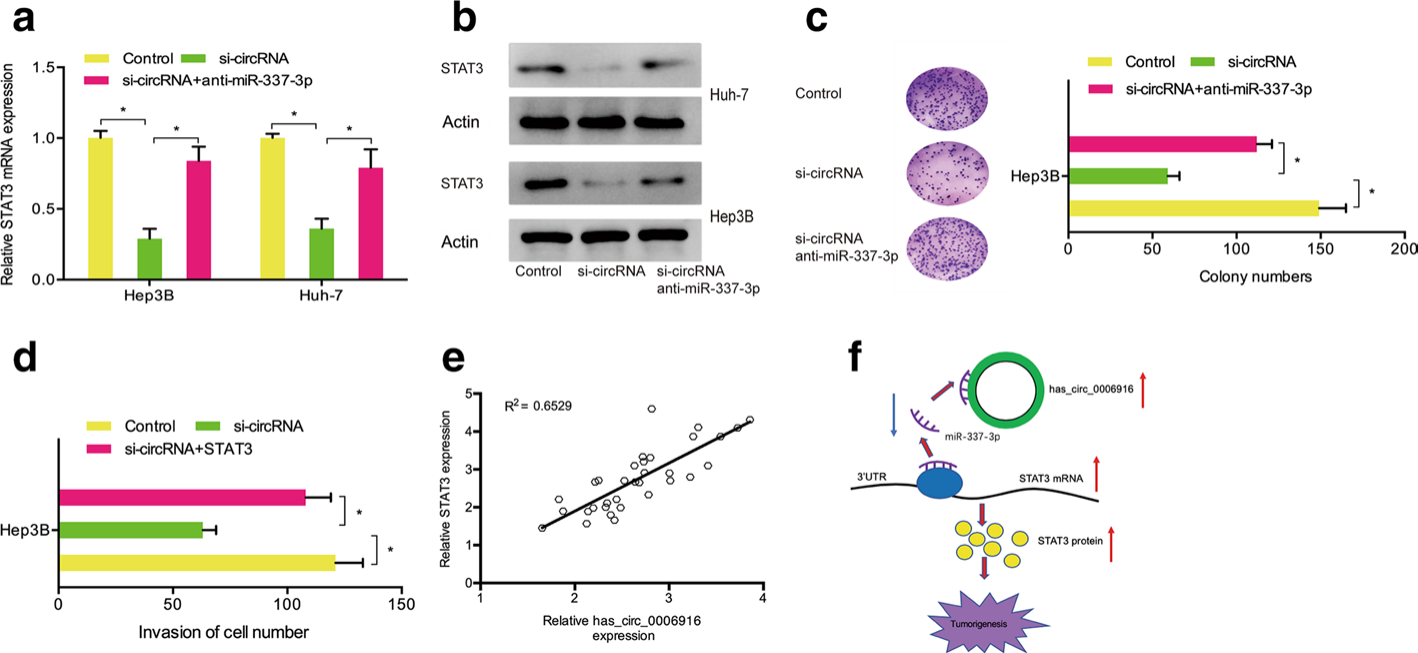
Hsa_circ_0006916/miR-337-3p/STAT3 axis in hepatocellular carcinoma. **a**, **b** miR-337-3p inhibitors rescued the effect of hsa_circ_0006916 suppression on STAT3 levels. **c**, **d** MiR-337-3p inhibitors rescued the effect of hsa_circ_0006916 silencing on growth and invasion of Hep3B cells. **e** Hsa_circ_0006916 is related to miR-337-3p in hepatocellular carcinoma. **f** The hsa_circ_0006916/miR-337-3p/STAT3 axis in hepatocellular carcinoma. *p < 0.05

Then, we determined whether hsa_circ_0006916 regulated hepatocellular carcinoma progression through the miR-337-3p/STAT3 axis using qRT-PCR and western blotting. The results demonstrated that hsa_circ_0006916 suppression reduced STAT3 levels in Hep3B and Huh-7 cells, whereas miR-337-3p inhibitors resolved the effects (Fig. 6a, b). Colony formation assay demonstrated that hsa_circ_0006916 silencing reduced Huh7 cell proliferation, while miR-337-3p inhibitors reversed the effects (Fig. 6c). Transwell assay showed that the number of invaded cells of the si-circ_0006916 group was significantly lower than that of the si-NC group, while STAT3 overexpression reversed the effect (Fig. 6d). Moreover, Spearman’s correlation analysis demonstrated a positive association between STAT3 and hsa_circ_0006916 expression in hepatocellular carcinoma (Fig. 6e). Therefore, these results suggest that hsa_circ_0006916 regulates hepatocellular carcinoma progression through regulating the miR-337-3p/STAT3 axis (Fig. 6f).

## Discussion

Recently, a number of circRNAs have been revealed to have a critical role in HCC progression. For example, Zhang et al*.* demonstrated that circRNA_104075 promotes YAP-dependent tumorigenesis in hepatocellular carcinoma through regulating HNF4a expression [16]. Wang et al*.* reported that circRHOT1 encouraged hepatocellular carcinoma progression by initiating NR2F6 [17]. Yu et al*.* showed that the circRNA cSMARCA5 decreased hepatocellular carcinoma cell growth and metastasis [18]. These findings suggest that circRNAs might act as potential therapeutic targets in hepatocellular carcinoma progression. In our study, we discussed the function of hsa_circ_0006916 in the development of hepatocellular carcinoma. Herein, we reveal the elevation of hsa_circ_0006916 levels in hepatocellular carcinoma samples and cell lines. High hsa_circ_0006916 levels in hepatocellular carcinoma were correlated with high TNM stage and lymph node metastasis. Loss-of-function experiments revealed that hsa_circ_0006916 exerted oncogenic functions by aggravating cell growth and invasion abilities.

Accumulating evidence has shown that circRNA serves as a competing endogenous miRNA and restores miRNA gene function [19]. In hepatocellular carcinoma, the ceRNA network modulated by circRNAs has been revealed by an increasing amount of studies [20, 21]. Herein, we discovered that hsa_circ_0006916 was mainly expressed in cytoplasm. Bioinformatics analysis showed that miR-337-3p has the hsa_circ_0006916 binding site. Moreover, RNA pull-down assay, luciferase report assay and qRT-PCR further validated the association between hsa_circ_0006916 and miR-337-3p, which indicates that hsa_circ_0006916 might regulate miR-337-3p levels in hepatocellular carcinoma. Previously, studies have indicated that downregulated miR-337-3p led to lower hepatocellular carcinoma cell proliferation and invasion abilities [22, 23]. Concordantly, we confirm that miR-337-3p showed low expression in hepatocellular carcinoma. Moreover, we validated the connection between hsa_circ_0006916 and miR-337-3p in hepatocellular carcinoma.

Next, we used bioinformatics to identify miR-337-3p’s possible target genes, and STAT3 was chosen as the candidate one. Previous studies have shown that STAT3 serves as an important regulator of tumorigenesis. For example, Yang et al*.* demonstrated that miR-218 suppressed lung cancer progression by targeting the IL-6/STAT3 axis [24]. Jang et al*.* reported that HOTAIR suppression in gastric cancer increased miR-454-3p to reduce tumor growth by the STAT3/Cyclin D1 axis [25]. Herein, we observed that expression of STAT3 was high in hepatocellular carcinoma tissues and cell lines. STAT3 knockdown reduced cell growth and invasive abilities in vitro. Moreover, we found that STAT3 overexpression reversed the tumor-suppressing ability of si-circ_0006916. Thus, we suggest that hsa_circ_0006916 overexpression promoted hepatocellular carcinoma progression through upregulating STAT3 expression as a miR-337-3p sponge.

In conclusion, our findings indicated a hsa_circ_0006916/miR-337-3p/STAT3 axis in hepatocellular carcinoma progression for the first time. We identified hsa_circ_0006916 as an oncogenic circRNA in hepatocellular carcinoma development, and provide a novel target for tumor treatment.

## Data Availability

The datasets supporting the conclusions of this article are included within the article.

## Abbreviations

ncRNAs: Non-coding RNAs
circRNA: Circular RNA
Wt: Wild-type
Mut: Mutant
QRT-PCR: Real-time quantitative PCR
EdU: 5-Ethynyl-2′-deoxyuridine
RIP: RNA immunoprecipitation
ceRNA: Competitive endogenous RNAs
HCC: Hepatocellular carcinoma
3′-UTR: 3′-Untranslated region
miRNA: MicroRNA
siRNAs: Short interfering RNAs
